# Supplementary material for: Potassium Lactate as a Strategy for Sodium Content Reduction without Compromising Salt-Associated Antimicrobial Activity in Salami
Source: Foods. 2021 Jan 7;10(1):114. doi: 10.3390/foods10010114 (PMC7826916; doi:10.3390/foods10010114)
Supplement: Supplementary file 1 [file foods-10-00114-s001.pdf]

Supplementary Figure 1

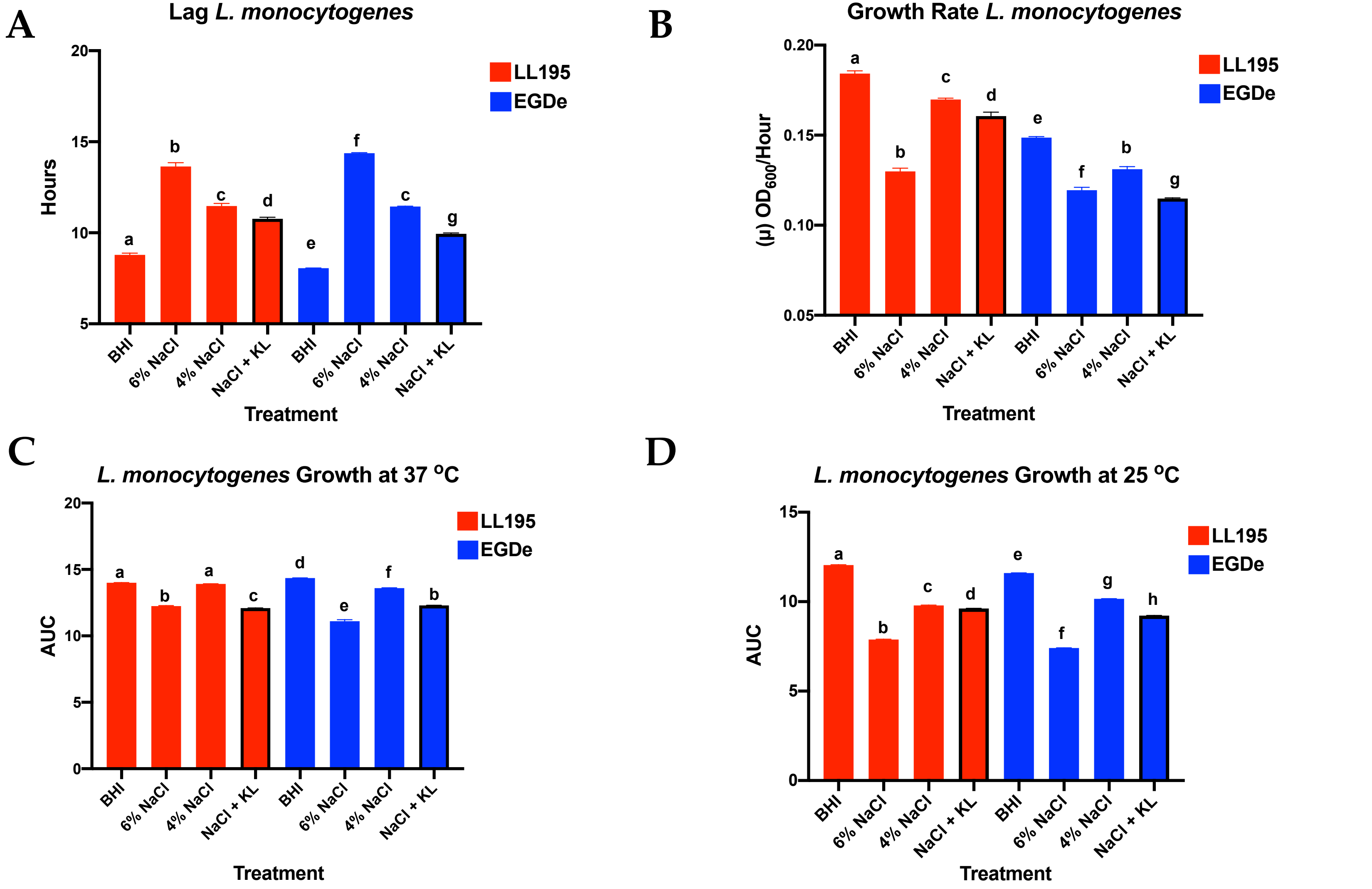

Figure S1 . The 2.8% sodium chloride and 1.6% potassium lactate (NaCl + KL) combination retains antimicrobial activity against *L. monocytogenes* (LL195 and EGDe) and temperature has a synergistic effect on the antimicrobial activity of both NaCl alone and the NaCl plus KL combination. (**A-B**) At 37 °C both interventions increase lag phase duration and reduce growth rate. (**C-D**) NaCl alone (6% and 4%) and the NaCl plus KL combination had a greater inhibitory effect at lower (25 °C vs 37 °C) temperature. Bars and error bars represent mean **(A)** lag phase duration **(B)** growth rate and (**C-D**) area under the curve generated using *opm* and GraphPad Prism from three biological replicates observed from kinetic growth assays in normal (BHI) and BHI supplemented with NaCl alone (6% and 4%) and the 2.8% NaCl plus 1.6% KL combination. Different letters indicate significant difference between strains ($P < 0.05$ based on one-way ANOVA and Tukey post-hoc test pairwise comparison of the treatments and strains).

Supplementary Figure S2

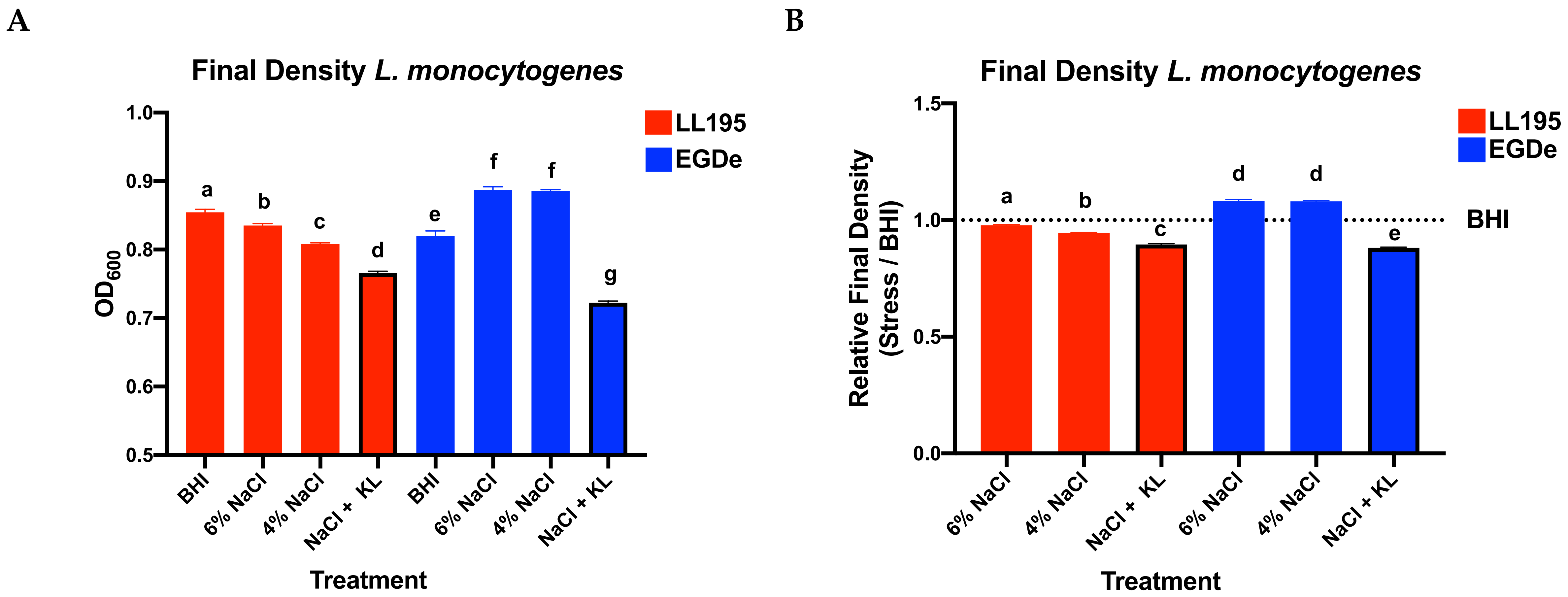

Figure S2. The reduced NaCl (2.8%) plus KL (1.6%) (NaCl + KL) combination retains antimicrobial activity as the high NaCl (4% and 6%) levels alone. Bars are (**A**) mean or (**B**) relative mean final bacteria cell density generated using *opm* from three biological replicates observed from kinetic growth assays at 37 °C in normal (BHI) as well as BHI supplemented with NaCl alone (4% and 6%) and the NaCl plus KL (NaCl + KL) combination. (**B**) Each parameter per treatment presented are mean final density expressed relative to those of the control with no stressor added, represented by the dotted line labeled BHI. Different letters indicate significant difference between treatments and strains ($P < 0.05$ based on one-way ANOVA and Tukey post-hoc test pairwise comparison of all the treatments).

Supplementary Figure S3

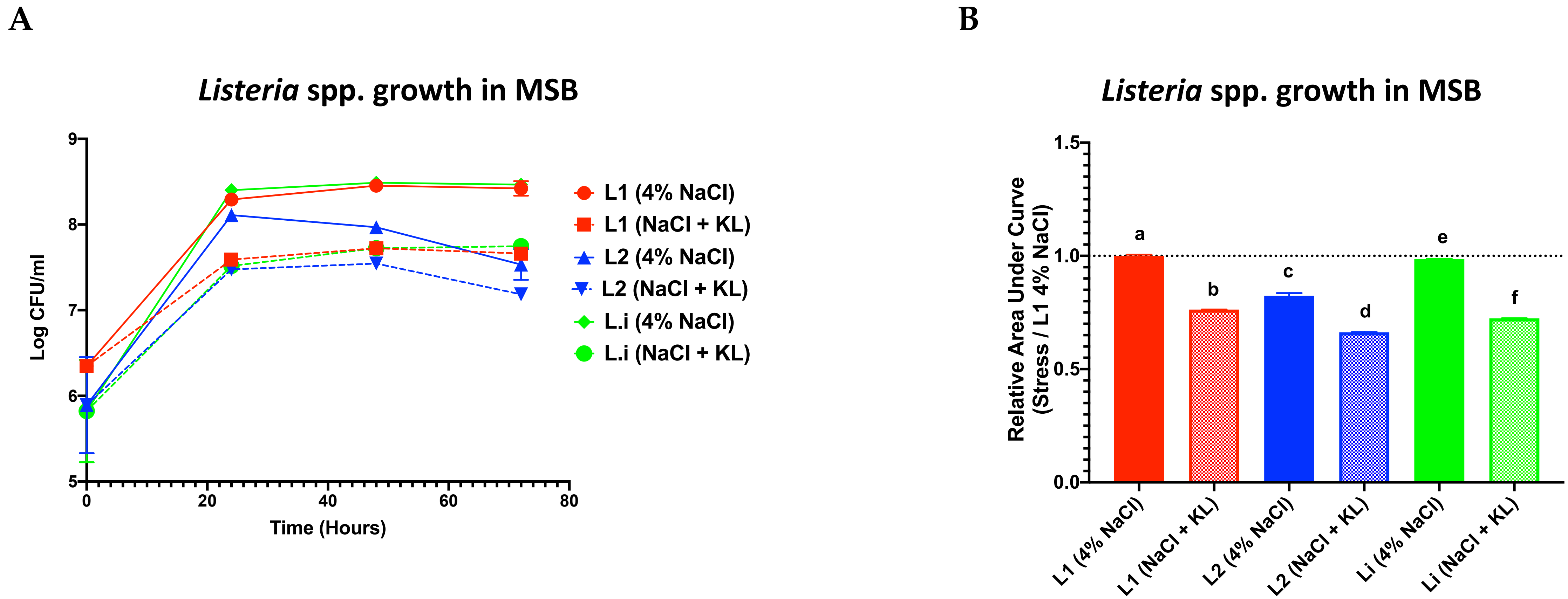

Figure S3. Comparison of strain cocktails of *L. monocytogenes* genetic lineage I (L1), lineage II (L2) and *L. innocua* (Li) growth in meat simulation broth (MSB) under stress associated with high NaCl concentration alone and the reduced NaCl plus KL combination. The 2.8% NaCl plus 1.6% KL combination (NaCl + KL) displays higher antimicrobial activity against the *L. monocytogenes* genetic lineage I and II as well as the *L. innocua* strain cocktails in comparison to the high salt (4% NaCl) concentration. Data presented is (**A**) growth profiles under simulated salami ripening conditions of the strain cocktails in MSB supplemented with 4% NaCl or 2.8% NaCl + 1.6% KL. **(B)** Bar graphs showing mean area under the curve (AUC) expressed relative to those of L1 NaCl, represented by the dotted line. Different letters indicate significant difference between treatments and strain cocktails ($P < 0.05$ based on one-way ANOVA and Tukey post-hoc test pairwise comparison).

Supplementary Figure S4

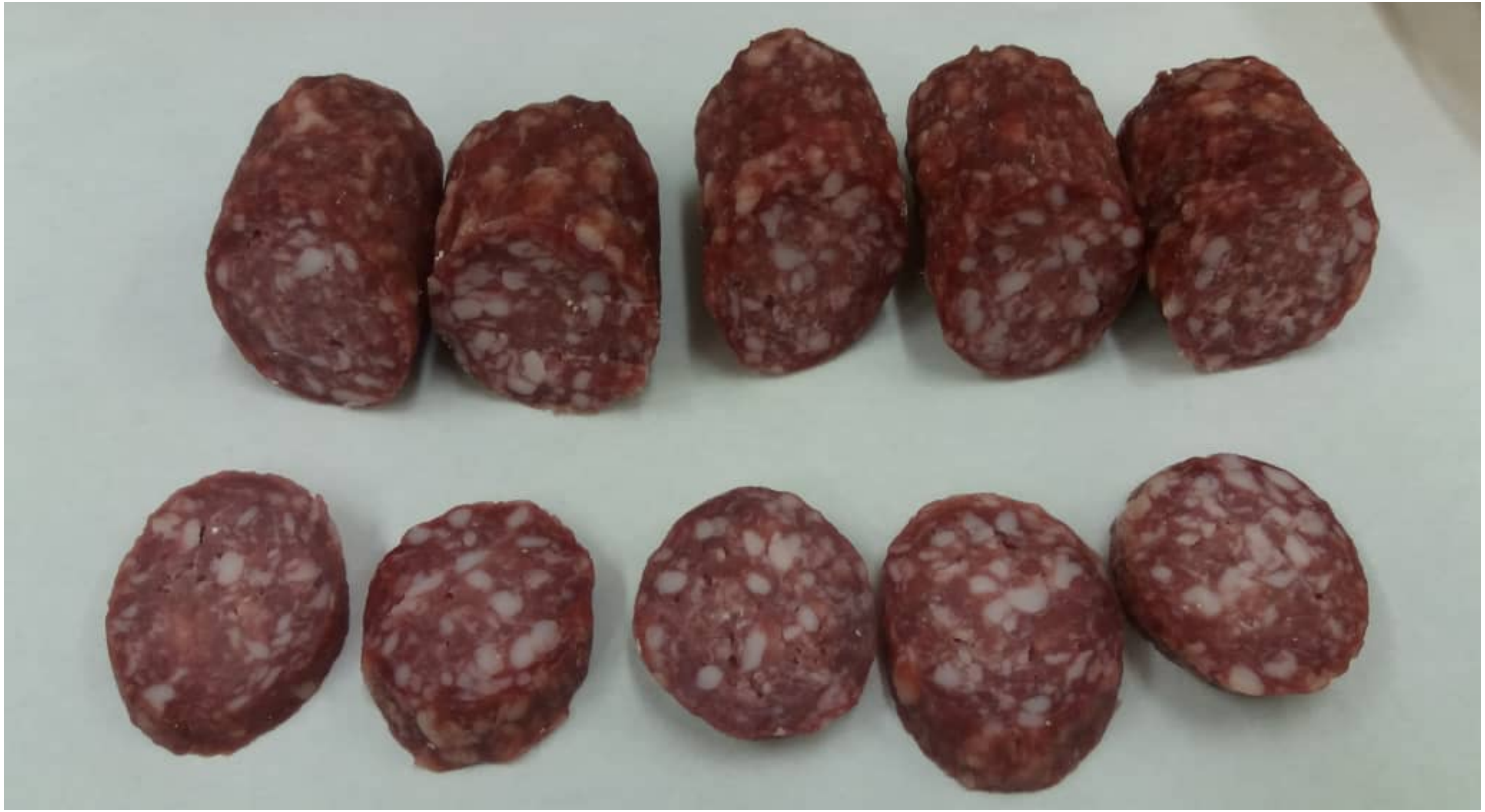

4% NaCl-no *Listeria* 4% NaCl 2.8% NaCl 2.8% NaCl + 1.6% KL 2% NaCl + 2.6% KL

Figure S4. Salami treated with different concentrations of NaCl alone and in combination with KL have similar color, texture and firmness.

Supplementary Figure 5

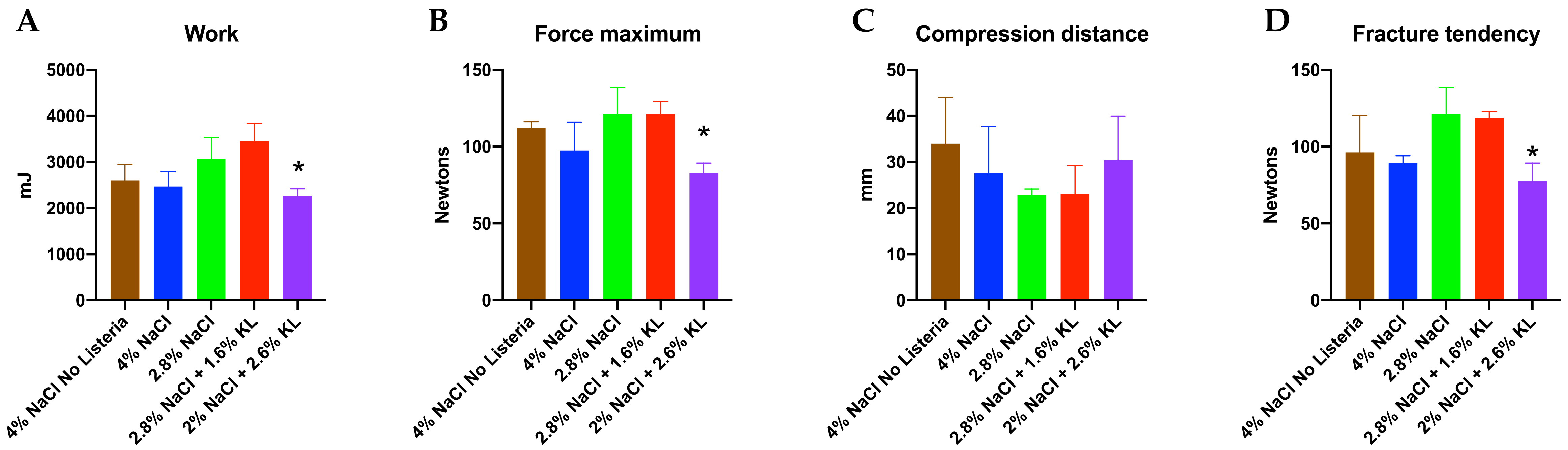

Figure S5. Comparison of salami firmness or tenderness based on Warner-Bratzler measurements. There were no significant differences in firmness or tenderness between salami treated with different concentrations of NaCl alone as well as the 2.8% NaCl plus 1.6% KL combination (30% NaCl reduction). Salami with 50% NaCl reduction (2% NaCl + 2.6% KL) displayed significant texture differences to the other salami based on the reduced energy (**A**) and force (**B**) required to cut through it.

Supplementary Tables:

Supplementary Table S1. Meat simulation broth composition

Ingredient	Amount
Glucose	5 g
Yeast extract	5 g
Bacto Tryptone	8 g
K_2HPO4	2 g
Tween 80	1 mL
Sodium chloride (NaCl)	50 g
MgSO4	0.2 g
MnSO4	0.015 g
Sodium lactate (50 %)	20 g
Distilled Water	912.085 mL

Supplementary Table S2. Salami reference recipe [a]

**Ingredients**	**Amount**	**Source**
Pork	6.5 kg	
Beef	1.5 kg	
Pork back fat	2.0 kg	
Salt (NaCl) [b]	260 g	
Ground white pepper	20 g	Omya Food, Balsthal, Switzerland
Garlic powder	10 g	Omya Food, Balsthal, Switzerland
Scheid-Bonafirm [c]	100 g	Scheid-Rusal AG, Gisikon, Switzerland
Starter culture SA1	Manufactures Rate	Agroscope, Bern, Switzerland

[a] Minced meat and fat were mixed with spices and starter culture or inoculum in a bowl mixer for 200 seconds. Salt was added and the mixture was mixed for another 100 seconds. [b] Reduced based on the target salt levels and recipe. [c] Contains: sugars (dextrose, maltodextrin, sucrose, lactose), acidifier (sodium citrate E 331), antioxidant (sodium ascorbate E 301, fatty acid ester of ascorbic acid E 304), preservative (potassium nitrate E 252).

Supplementary Table S3. Salami production conditions [a]

Steps	Temperature (°C)	Relative humidity (%)	Time (h)	Cumulative time (h)
0[b]	24	96	48	0
1	24	96	48	48
2	22	90	48	96
3	20	85	48	144
4	18	82	48	192
5	18	78	48	240
6	15	74	48	288
7	15	72	192	480

[a] Salami ripening chamber conditions maintained following Agroscope Switzerland salami ripening protocol recommendations. The salami sausages were dried until at least 30 % weight loss. [b] Ripening chamber temperature and humidity acclimatization phase.

Supplementary Table S4. Comparison of *L. monocytogenes* EGDe and LL195 growth in BHI at 37 °C under NaCl and potassium lactate (KL) stress[a]

	EGDe			
Condition	**Growth rate** μ (OD_{600}/hr)	**Lag phase duration (hr)**	**Final cell density (OD_{600})**	**AUC[b]**
BHI	0.149 ± 0.001	8.058 ± 0.007	0.820 ± 0.008	11.602 ± 0.104
4% NaCl	0.131 ± 0.001	11.446 ± 0.015	0.886 ± 0.002	10.156 ± 0.022
6% NaCl	0.119 ± 0.002	14.373 ± 0.027	0.887 ± 0.004	7.407 ± 0.049
2.8% NaCl + KL 1.6%	0.115 ± 0.000	9.942 ± 0.049	0.722 ± 0.003	9.215 ± 0.042

	LL195			
**Condition**	**Growth rate** μ (OD_{600}/hr)	**Lag phase duration (hr)**	**Final cell density (OD_{600})**	**AUC[b]**
BHI	0.184 ± 0.001	8.791 ± 0.092	0.854 ± 0.005	12.051 ± 0.077
4% NaCl	0.170 ± 0.001	11.476 ± 0.134	0.808 ± 0.002	9.789 ± 0.071
6% NaCl	0.130 ± 0.002	13.641 ± 0.208	0.835 ± 0.003	7.880 ± 0.123
2.8% NaCl + KL 1.6%	0.161 ± 0.001	10.766 ± 0.091	0.765 ± 0.003	9.616 ± 0.095

[a] Presented values are means and their standard deviations generated using *opm* from three biological replicates observed from kinetic growth assays (24 hrs, 37 °C) in normal (BHI only) as well as BHI supplemented with 4% and 6% NaCl as well as the NaCl (2.8%) plus KL (1.6%) combination. [b] AUC: area under the curve.

Supplementary Table S5. Comparison of *L. monocytogenes* and *L. innocua* growth in BHI at 25 °C under NaCl and KL stress

	Ander under the Curve [a]					
**Growth Condition**	**LL195**	**EGDe**	***L. inocua* 20869**	***L. inocua* 20870**	***L. inocua* 20871**	***L. inocua* 20872**
**BHI only**	16.03 ± 0.02	15.86 ± 0.04	19.34 ± 0.02	18.09 ± 0.04	18.62 ± 0.03	19.62 ± 0.03
**4% NaCl**	12.28 ± 0.04	12.13 ± 0.01	14.68 ± 0.02	13.91 ± 0.04	14.12 ± 0.06	14.76 ± 0.08
**2.8% NaCl + 1.6% KL**	11.33 ± 0.03	11.02 ± 0.04	14.2 ± 0.07	12.8 ± 0.08	13.18 ± 0.1	14.46 ± 0.13

[a] Presented values are the mean area under the curve generated using GraphPad Prism from three biological replicates observed from kinetic growth assays (48 hrs) in normal (BHI only) as well as BHI supplemented with 4% NaCl and the NaCl (2.8%) plus KL (1.6%) combination.

Supplementary Table S6. Comparison of growth relative to 4% NaCl treated salami [a]

Time (days)	2.8% NaCl	2.8% NaCl + 1.6% KL	2% NaCl + 2.6% KL
0	0	0	0
1	0.004	-0.117	-0.150
2	0.110	0.002	-0.070
4	0.097	-0.026	-0.129
7	0.258	0.026	0.080
9	0.177	0.098	0.028
13	0.253	0.186	0.017
16	0.099	0.200	0.135
20	0.356	0.244	0.111
27	0.131	-0.036	-0.170
64	0.205	-0.127	-0.297
144	0.897	-0.591	-0.778

[a] Presented value are calculated as log CFU/sample/day minus 4% NaCl treated salami log CFU/day. Negative values correspond to lower log CFU count in comparison to 4% NaCl treated salami counts on that day.

Supplementary Table S7. Comparison of growth relative to day zero inoculum [a]

Time (days)	4% NaCl	2.8% NaCl	2.8% NaCl + 1.6% KL	2% NaCl + 2.6% KL
0	0	0	0	0
1	0.119	0.123	0.002	-0.031
2	-0.172	-0.062	-0.170	-0.242
4	-0.222	-0.125	-0.248	-0.350
7	-0.215	0.043	-0.189	-0.135
9	-0.170	0.007	-0.072	-0.142
13	-0.135	0.118	0.050	-0.118
16	-0.136	-0.037	0.064	-0.001
20	-0.358	-0.002	-0.113	-0.246
27	-0.179	-0.048	-0.216	-0.350
64	-0.196	0.009	-0.323	-0.492
144	-1.144	-0.247	-1.734	-1.922

[a] Presented value are calculated as log CFU/sample/day minus day zero inoculum log CFU counts. Negative values correspond to lower log CFU count in comparison to day zero inoculum counts.

Supplementary Table S8. Degree-hours calculation [a]

The pH dropped below pH 5.3 within 48 hours of fermentation at 24 °C.

Degrees above 15.6 °C: 24 °C - 15.6 °C = 8.4 °C

Hours to reach pH 5.3: 48 hrs

Degree-hours calculation: 8.4 °C × 48 = 403.2 degree-hours

The corresponding degree-hours limit (24 °C) is 665 degree-hours.

[a] Calculation derived from: Preventive control recommendations for manufacturing fermented and dried meat products (https://www.inspection.gc.ca/preventive-controls/meat/fermented-and-dried/eng/1522951036924/1522951037158#a1) accessed 26th November 2020.
